# Tuning the Interfacial Deformation of Gliadin-Flaxseed Gum Complex Particles for Improving the Foam Stability

**DOI:** 10.3390/gels10110677

**Published:** 2024-10-22

**Authors:** Ping Wu, Wei Shang, Jiaqi Shao, Qianchun Deng, Jisong Zhou, Xia Xiang, Dengfeng Peng, Weiping Jin

**Affiliations:** 1 Key Laboratory of Oilseeds Processing, Ministry of Agriculture, Oil Crops Research Institute, Chinese Academy of Agricultural Sciences, Wuhan 430062, China; wuping6868@163.com (P.W.);; 2 School of Food Science and Engineering, Wuhan Polytechnic University, Wuhan 430023, China

**Keywords:** gliadin, flaxseed gum, particle flexibility, gel-like network structure, air/water interface, Lissajous plots, foam stability

## Abstract

Gliadin nanoparticle (GNP) is a promising foaming agent, but its application is hindered by the limited foam stability under low acidic conditions. Herein, we attempted to tune the foam stability of GNP by coating it with flaxseed gum (FG) and investigated the structure, interfacial behaviors, and foam functionality of gliadin-FG (GFG) particles at pH 4.5. Results showed that the formation of GFG complex particles was driven by an electrostatic interaction between positive charge patches on the surface of GNP (~17 mV) and negative charges in FG molecule (~−13 mV) at all tested ratios. The addition of appropriate amounts of FG (1:0.05) effectively improved the foam stability of GNP. This was because GFG with larger sizes and lower surface charge possessed higher rigidity after coating with FG. When they adsorbed at the air/water interface, their deformation process was slower than that of GNP, as indicated by interfacial dilatational rheology and cryo-SEM, and the covered particles seemed to be more closely distributed to form solid-like and dense interfacial films. Notably, the addition of FG at a higher ratio (1:0.3) promoted the foam stability of GNP by about five folds because the larger GFG with suitable flexibility and wettability could form a stiff interface layer with more significant elastic response, and the unabsorbed particles and FG could form a gel-like network structure in the continuous phase. These characteristics effectively prevented foam disproportionation and coalescence, as well as retard the drainage. Our findings demonstrate that coating GNPs with FG is an effective approach to improve their application in foamed foods.

## 1. Introduction

Aqueous foam is an essential ingredient in many foods (such as cake, ice cream, and beer), which can significantly improve the color, texture, and overall sensory quality of the products [1,2]. Aqueous foam is a sub-stable system, whose formation and stabilization heavily depend on the properties of the foaming agents [3]. Particles are one of the desirable foaming agents due to their irreversible adsorption at the air/water interface [4,5]. Food-grade particles have become a popular foam stabilizer in foamed foods, as they can endow strong stability to the foams through the Pickering mechanism [6,7]. Recently, many types of biomacromolecules have been used to fabricate food-grade particles, such as water-soluble protein [8], prolamin [9,10], and polysaccharide [11,12].

Gliadin belongs to prolamin, which possesses a terminal structural domain that is more hydrophobic than the central structural domains, and it exhibits tunable self-assembly behaviors [13]. These structural advantages make gliadin an ideal candidate to form colloidal particles with desirable surface properties, which confers it with superior foaming ability, good foam plasticity, and high foam stability [14]. However, gliadin nanoparticles (GNPs) are highly susceptible to pH, and their foam stability begins to decline when the pH is lower than 5 [2]. To expand its application in aerated foods, there is an urgent need for effective strategies to enhance its foam stability at low acidic conditions.

Polysaccharides have wide applications in biomedical and food fields [15]. Recent studies have demonstrated that polysaccharides can promote the foaming or emulsifying properties of GNP by regulating their interfacial behaviors [16,17]. For example, it has been shown that pectin can effectively modulate the arrangement of GNP at the air/water interface, thereby resulting in high foam stability over a wide pH range [14]. In addition, the interaction of soybean polysaccharides with gliadin to form composite particles resulted in the formation of a thicker interfacial film at the oil/water interface, which significantly improved the stability of the emulsion [18]. Our previous research has indicated that GNP can be deformed at the interface, which has a great impact on its interfacial properties and will affect its foam stability [19]. However, it remains unclear how polysaccharides affect the interfacial deformation of GNP and then regulate their foam stability. Flaxseed gum (FG), derived from flaxseed (*Linum usitatissimum* L.), is a negatively charged natural hetero-polysaccharide that is environmentally sustainable, low-cost, biocompatible, and biodegradable. In addition, as a hydrocolloid, it has high viscosity as well as good foaming, gelling, and emulsifying properties [20]. It can interact with proteins and influence their structures and interfacial properties, thus leading to the promotion of emulsifying properties [21]. Hence, it is meaningful to investigate the influence of FG on the interfacial deformation and foam stability of GNP.

This study investigated how the addition of FG at different ratios affects the structural, interfacial, and foaming properties of GNP. We first examined the physical properties of the complex particles (GFG), mainly including turbidity, viscosity, particle size, zeta potential, morphology, flexibility, and surface wettability. Then, we investigated the rheological properties and morphology of GFG at the air/water interface as a function of time. Subsequently, we evaluated the foaming functionalities of GFG. Finally, we attempted to build the link among the above properties to explore the possible foam stabilization mechanism. This study provides an effective approach to enhance the foam stability and application potential of GNP.

## 2. Results and Discussion

### 2.1. Structural Properties of Gliadin-Flaxseed Gum Particles

#### 2.1.1. Appearance, Turbidity, and Viscosity

**Figure 1:**
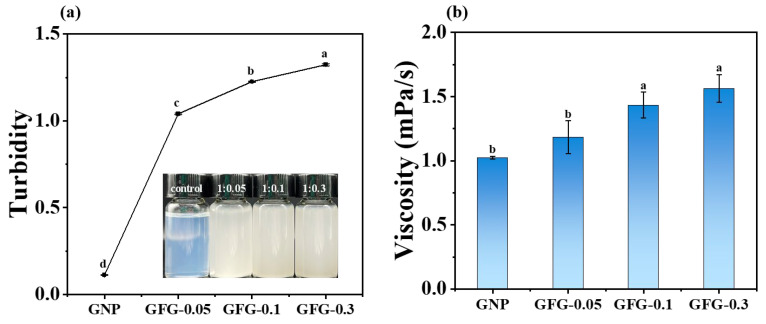
Turbidity (**a**) and viscosity (**b**) of GNP and GFG. The insert in (**a**) is the visual appearance of the samples (Note: GFG-0.05, GFG-0.1, and GFG-0.3: gliadin/FG added at a ratio of 1:0.05, 1:0.1, and 1:0.3 (*w*/*w*), respectively. The same in the figure below). Different letters such as a, b, c, d in Figure 1a and Figure 1b indicate significant differences (*p* < 0.05).

Figure 1a exhibits the appearance and turbidity of GNP and GFG. GNP showed a light blue color at pH 4.5 with low turbidity (~0.11). The addition of FG at different ratios significantly increased the turbidity of particles, which might be due to the formation of larger complex particles from the interaction between FG and GNP. The increase in turbidity became more pronounced with increasing the amount of additional FG. Figure 1b shows the viscosity of GNP and GFG. GFG prepared with the addition of FG at ratios of 1:0.1 (GFG-0.1) and 1:0.3 (GFG-0.3) showed higher viscosity than GNP, particularly GFG-0.3, whose viscosity was 1.5-fold than that of GNP, indicating that the viscosity of the suspensions can be regulated by FG.

#### 2.1.2. Particle Size, Zeta Potential, and Morphology

**Figure 2:**
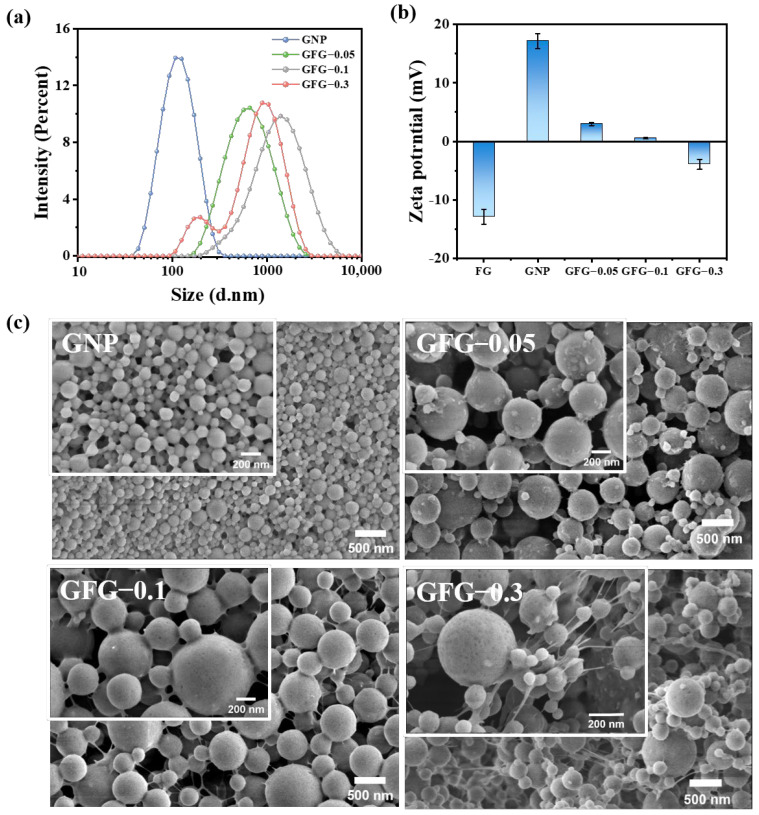
Particle size (**a**), zeta potential (**b**), and SEM images (**c**) of GNP and GFG with the addition of various amounts of FG.

The particle size, surface charge, and morphology of particles are key factors influencing their interfacial and foaming properties [6,22]. Figure 2 shows the above structural properties of GNP and GFG. The particle size of GNP was close to 100 nm (Figure 2a), which belongs to the category of nanoparticles [23], similar to the results reported by Zhu et al. and Peng et al. [14,24]. The particle sizes of all investigated GFG-based samples obviously increased after the addition of FG (Figure 2a). Among them, GFG-0.1 showed nearly monomodal distribution and the largest particle size, whereas the particle size distribution of GFG-0.3 had two peaks, indicating that they had a relatively non-uniform size distribution. Figure 2b shows the charge of samples. FG is a negatively charged anionic polysaccharide [25] with a zeta potential at pH 4.5 of ~−13 mV, while GNP displayed a positive charge of ~17 mV at pH 4.5. They could self-assemble to form complex particles with larger sizes via electrostatic interaction. The addition of FG obviously altered the zeta potential value of GNP. When FG was added at a ratio of 1:0.1 (*w*/*w*), the absolute value of zeta potential was close to 0 mV. At this time, the surface charge of the GFG was low and the interaction among particles strengthened, leading to an increase in particle size.

The microstructures of GNP and GFG are shown in Figure 2c. Both GNP and GFG displayed spherical-like particles. GNP exhibited the smallest particle size and uniform distribution, while the addition of FG significantly increased the particle size. This further demonstrated the formation of complex particles. GFG-0.1 seemed to possess the highest particle size, and GFG-0.3 showed an uneven size distribution. These corresponded to the DLS results (Figure 2a). Interestingly, the filamentous gel-like continuum between spherical particles occurred with increasing FG, which might result in the increase of suspension viscosity (Figure 1b). Particularly for GFG-0.3, the gel-like structures were much more obvious than that of GFG-0.1, and the particles seemed to be entangled by these gel-like structures. This also corresponded to the higher viscosity of GFG-0.3.

#### 2.1.3. Flexibility and Surface Wettability

**Figure 3:**
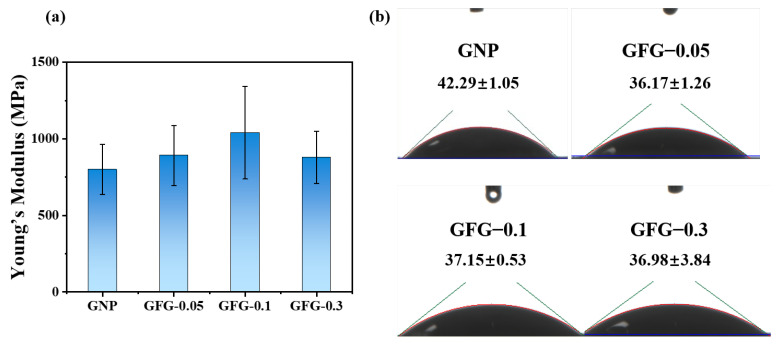
Young’s modulus (**a**) and contact angle (**b**) of GNP and GFG with the addition of various amounts of FG.

The flexibility and surface wettability of particles are also important factors influencing their interfacial properties [26,27]. Here, the flexibility and surface wettability of GNP and GFG are assessed using Young’s modulus obtained from AFM and contact angle, respectively, and the results are shown in Figure 3. The rigidity of GFG increased first and then decreased. This illustrated that FG could tune the mechanical strength of particles. When the FG addition ratio was 1:0.1 (*w*/*w*), it reached the maximum (Figure 3a). These indicated that the flexibility of GNP can be effectively regulated by coating with FG. At the air/water interface, the hydrophobic surfaces are characterized by a contact angle greater than 90°, while hydrophilicity is dominant when the contact angle is smaller than 90° [28]. GNP and GFG with different ratios had contact angles smaller than 90° and thus showed hydrophilic surfaces. GNP possessed the largest contact angle (42.29°), whereas the addition of FG decreased the contact angle of GNP, illustrating that GFG showed more hydrophilicity (Figure 3b). This might be because FG was more hydrophilic, and when it was coated on the surface of GNP, it could endow more hydrophilic groups on the surface of complex particles.

### 2.2. Interfacial Properties of Gliadin-Flaxseed Gum Particles

#### 2.2.1. Interfacial Adsorption Kinetics

**Figure 4:**
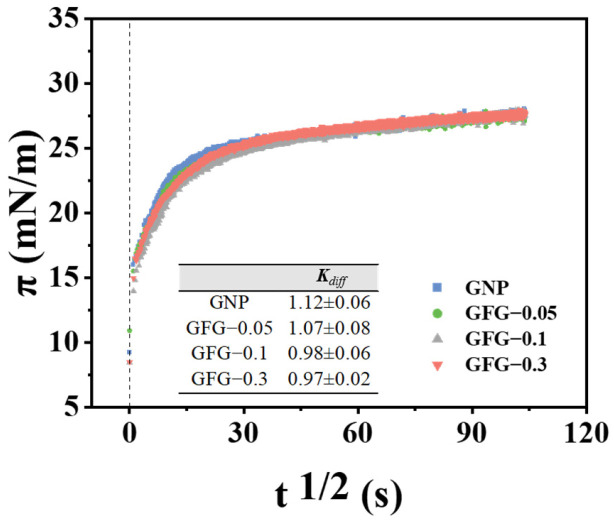
Curves of surface pressure (*π*) versus *t*^1/2^ for GNP and GFG at the air/water interface. The insert table is *K*_diff_.

The interfacial adsorption process of particles is closely associated with their foam properties [29,30]. The adsorption of soft particles at the interface can be categorized into adsorption, conformational change, spread, and structural rearrangement [31]. Figure 4 shows the surface pressure (*π*) as a function of *t*^1/2^ for GFG and GNP. It can be observed that the initial adsorption process of GFG and GNP at the air-water interface was linear, indicating that this process was dominated by diffusion, and the slope of *π*-*t*^1/2^ curve (*K*_diff_) could be used to assess the adsorption rate of the particles in the initial phase [32]. Based on the insert table in Figure 4, the *K*_diff_ value of GNP seemed to be slightly larger than that of GFG-0.1 and GFG-0.3, indicating that GNP had a faster initial adsorption rate. This might be related to its higher surface wettability and smaller particle size [33]. Notably, despite an increase in particle size and a concomitant decrease in wettability, GFG-0.1 and GFG-0.3 exhibited rapid adsorption rates. This phenomenon might be attributed to the suitable flexibility and lower surface charge, which reduced the energy barrier of GFG in the initial adsorption phase. After the rapid adsorption stage, the *π* value of GFG and GNP began to rise slowly until reaching a relative equilibrium value at 10,800 s (~27.7 mN/m). These phases were closely related to the conformational change, spread, and rearrangement of particles.

#### 2.2.2. Interfacial Dilatational Rheology

**Figure 5:**
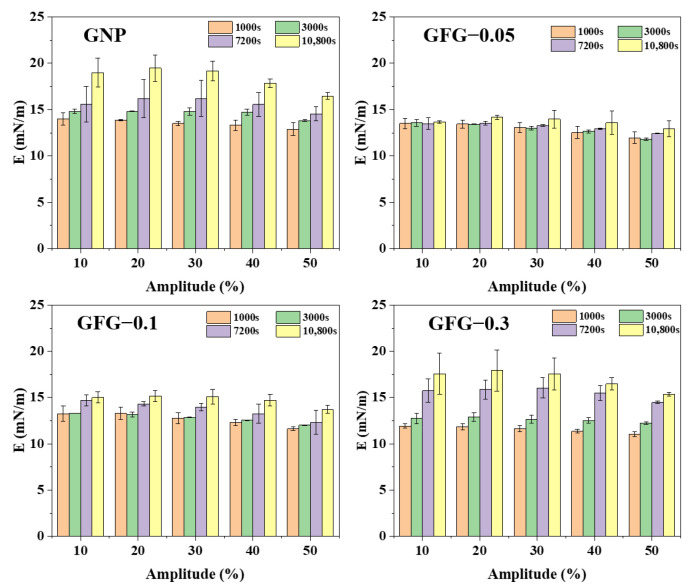
Variation of viscoelastic modulus (*E*) of GNP and GFG at amplitude sweeps (10–50%) after different start oscillation times (1000 s, 3000 s, 7200 s, and 10,800 s).

The rheological properties of the GFG- and GNP-stabilized interfaces were investigated using dilatational amplitude sweeps (10–50%) rheology [34]. Adsorption was performed for 1000, 3000, 7200, and 10,800 s, and the results of viscoelastic modulus (*E*) are presented in Figure 5. It was worth noting that the interfaces were not dilated during the adsorption process. For GNP and GFG-0.3 at amplitudes of 10%, 20%, 30%, 40%, or 50%, the *E* value showed a clear increasing tendency with the extension of adsorption time. These results implied that the interfacial structures of GNP continuously varied over adsorption time due to non-destructive behavior during adsorption. In contrast, as a function of adsorption time (1000–10,800 s), the *E* values of GFG-stabilized interfaces with FG addition ratios of 1:0.05 and 1:0.1 remained the same or increased slightly at different amplitudes, indicating that the interfacial structures of GFG-0.05 and GFG-0.1 showed no evident change along with adsorption [35]. These results suggested that the addition of an appropriate amount of FG could delay the change in the interfacial structure of GNP.

**Figure 6:**
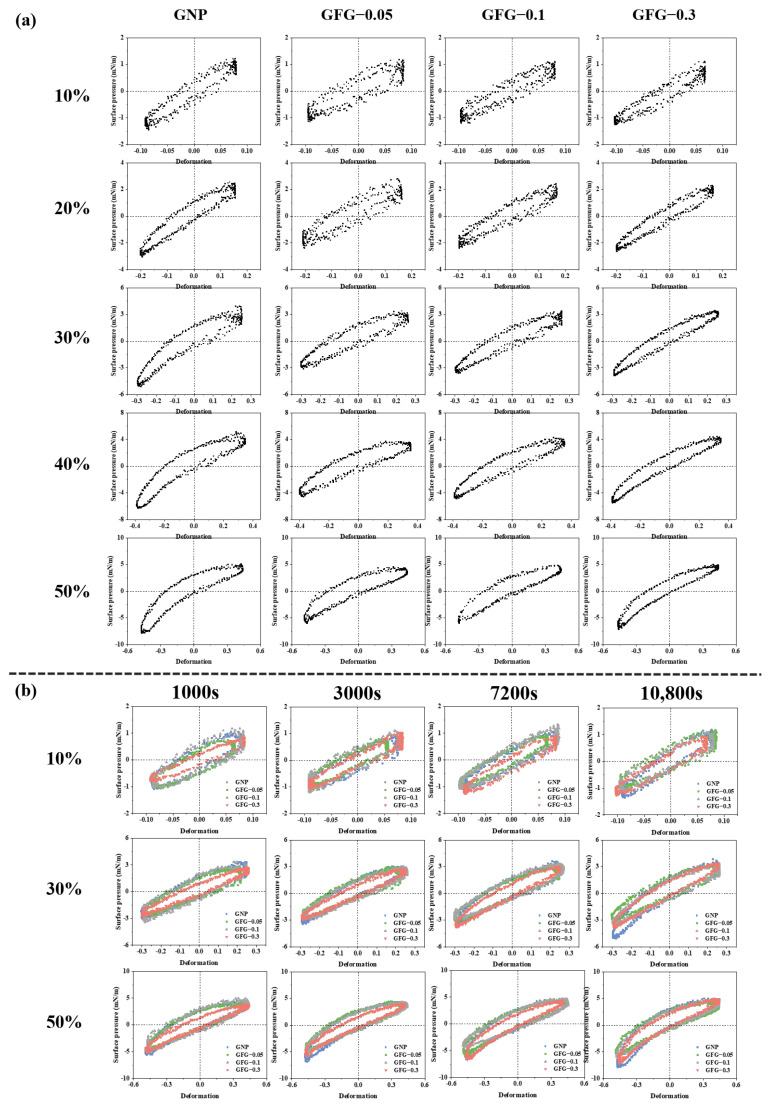
Lissajous plots of GNP and GFG obtained during amplitude sweeps (10–50%) after adsorption of 10,800 s (**a**), and Lissajous plots at amplitudes of 10%, 30%, and 50% after adsorption times from 1000 s to 10,800 s (**b**).

To further elucidate the relation between variations in amplitude sweep and the structure and mechanical behavior of the interfacial layer, as well as the interaction between adsorbed particles, we generated Lissajous plots to depict the surface pressure against deformation [35,36]. The plots are displayed in Figure 6. GNP- and GFG-formed interfaces had an ellipsoidal shape, implying that both of them had viscoelastic solid-like properties. At a deformation rate of 10%, and after adsorbing for 10,800 s, the GNP- and GFG-stabilized interfaces at different ratios exhibited a narrow and approximately symmetric ellipsoid shape (Figure 6a), indicating that elastic response was predominant. Subsequently, the Lissajous plots gradually became asymmetric with larger deformations, suggesting progressive disruption of the microstructure of interfacial film with increasing amplitude, in which the viscous component gradually became dominant [37]. At the amplitude of 50%, the above phenomenon became more pronounced. For the extension process (the upper part of the loop) of the GNP- and GFG-stabilized interfaces, the surface pressure first showed a sharp increase at the initial stage, indicating an elastic-dominant response. Subsequently, the slope of the surface pressure gradually decreased, implying an intra-cycle strain softening behavior [38,39]. For the compression process (the lower part), the values of surface pressure of the GNP- and GFG-stabilized interfaces were higher than those for the extension process, implying an intra-cycle strain hardening behavior, probably due to the jamming of adsorbed particles [40]. Based on the above analysis, it can be speculated that GNP showed a stronger in-plane interaction and a stiffer interfacial structure. In contrast, GFG-0.05 and GFG-0.1 had less strain softening upon extension and less strain hardening upon compression. These results indicated that they might have a slightly weaker in-plane interaction, but the interfaces stabilized by GFG-0.05 and GFG-0.1 still showed a viscoelastic solid-like structure. The GFG-0.3-stabilized interface had a notably narrower cycle than that of GNP, suggesting that GFG-0.3 can fabricate cohesive and stiff interfaces with a more elastic response.

Additionally, we also constructed the Lissajous plots of GNP- and GFG-formed interfaces at various amplitudes (10%, 30%, and 50%) after different adsorption times (1000, 3000, 7200, and 10,800 s), as shown in Figure 6b, with the aim to discover the changes in mechanical behavior and particle interactions over adsorption time. At a fixed amplitude of 10%, GNP- and GFG-stabilized interfaces at different adsorption times displayed an approximately symmetric ellipsoidal shape, indicating that both of them had viscoelastic solid-like properties. With the extension of adsorption time from 1000 s to 10,800 s, all the loops seemed to be more tilted towards the vertical axis, implying that the interfaces stabilized by GNP and GFG at different ratios became slightly stiffer with the adsorption time. When the fixed amplitude increased to 50%, all the interfaces confined by GNP and GFG showed strain softening in extension and strain hardening in compression at the investigated adsorption time, indicating the microstructure disruption of interfacial layers [37]. At different adsorption times, such as 1000 s and 10,800 s, the variation trend of loop shapes for different samples was different. The interfaces stabilized with GNP and GFG-0.3 at 10,800 s had a more extensive phenomenon than that at 1000 s, with regard to strain softening in extension and strain hardening in compression. This further illustrated that the interfacial structures of GNP and GFG-0.3 were continually transforming over adsorption time. Interestingly, the interfaces stabilized by GFG-0.05 and GFG-0.1 showed a relatively slow trend of loop shape from 1000 s to 10,800 s, suggesting that the addition of suitable amounts of flaxseed gum could restrain the changes of the interfacial structure of GNP.

#### 2.2.3. Interfacial Morphology of Foams

**Figure 7:**
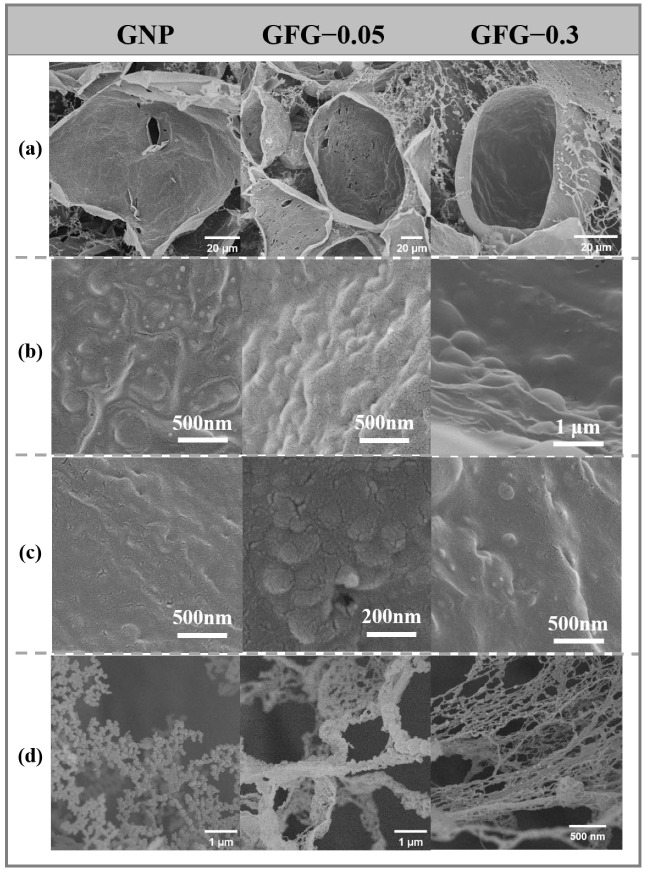
Cryo-SEM images of GNP- and GFG-covered foam (**a**), foam surface after storing for 1.5 min (**b**), foam surface after storing for 30 min (**c**), and continuous phase of foam systems (**d**).

To further illustrate the change of microstructure with regard to adsorbed GNP and GFG, cryo-SEM is used to capture their morphology at different storage times, and the images are shown in Figure 7. At a store time of 1.5 min, the surface layer stabilized using GNP, and GFG was covered by the observable particles (Figure 7b), suggesting that both GNP and GFG could speedily adsorb at the air/water interface. This was accordant with the adsorption results in Figure 4. Compared with the GNP-stabilized surface, the GFG-confined one had larger visible particles and were more closely distributed, therefore, they seemed to be denser. As the storage time increased to 30 min, the surface layers covered by GNP and GFG-0.3 had reduced visible particles. This might be related to the particle deformed along the interface and the interaction among adsorbed particles. Compared with the surface layers formed by the two particles, the one covered by GFG-0.3 seemed to have more observable deformed particles, suggesting that the deformation rate of GFG-0.3 was slower than that of GNP. Surprisingly, the surface stabilized by GFG-0.05 remained the most visible particles and appeared to be denser. These results indicated that the coating of FG could slow down the deformation process of GNP, which might be related to the rigidity of particles. In addition, the continuous phase images of foam systems formed by GNP and GFG were different (Figure 7d). For the GNP-stabilized foam, the continuous phase included the unabsorbed GNPs. Interestingly, the addition of FG caused the unabsorbed particles to form a gel-like network structure under the entanglement of FG. GFG-0.3 produced a more compact gel-like network structure in the continuous phase. This increased the viscosity of the continuous phase.

### 2.3. Foaming Properties of Complex Particles

**Figure 8:**
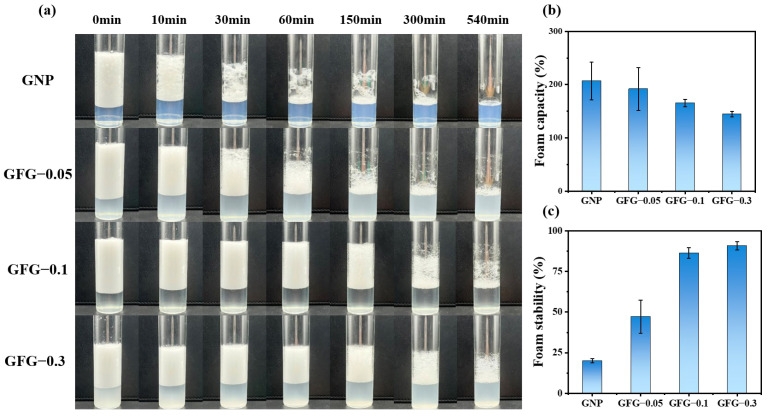
Foam visual appearance (**a**), foam capacity (**b**), and foam stability (**c**) of GNP and GFG with the addition of various amounts of FG.

Figure 8 displays the foam appearance (from 0 min to 540 min), foam capacity, and foam stability for GNP and GFG at a pH of 4.5. The foaming capacity of GNP and GFG-0.05 could reach about 200%, which was significantly higher than that of the soybean isolate protein (about 80%) [41] and whey protein (lower than 120%) at the same pH [42]. Compared with the GNP, GFG-0.1 and GFG-0.3 had a slightly lower foam capacity, which might be due to the reduced adsorption rate. With the additional increase of flaxseed gum, the foam stability showed a gradual increase from 20% to 90%. Basically, foam stability is usually affected by drainage, disproportionation, and coalescence [43,44]. Here, GFG-0.3 possessed the highest foam stability. This could be because, on one hand, the continuous phase of a GFG-0.3-based foam system showed a gel-like network structure, which could reduce the drainage rate and improve the foam stability [45,46]. On the other hand, the adsorbed GFG-0.3 could form a dense and stiff interfacial layer with a more elastic response and slower deformation rate, which could effectively prevent foam disproportionation and coalescence.

## 3. Conclusions

In this study, we explored the effect of FG with the different ratios on the structural, interfacial, and foam properties of GNP. Three major significations were obtained. First, the complex particles could be formed by coating FG on GNP, mainly via an electrostatic interaction. Second, the addition of appropriate amounts of FG with a ratio of 1:0.05 could increase the size and rigidity, as well as decrease the surface charge of GNP. These structural properties enabled the particles to be quickly absorbed, slowly deformed, and closely distributed at the interface, and thus formed solid-like and dense interfacial films. This might improve its foam stability. Third, when the addition ratio of FG was 1:0.3, the complex particle could form a stiff interface layer with a more elastic response and slower deformation rate, and the gel-like network structure became more compact in its continuous phase. These characteristics endow it with the highest foam stability and broaden its application in foam products. These results provide some theoretical basis for expanding the application of gliadin particles in foamed products.

## 4. Materials and Methods

### 4.1. Materials

Gliadin with a purity of 80.6% was extracted from gluten as described by Peng et al. [14]. Flaxseeds (Zhang Ya 2#) were purchased from the Gansu Academy of Agricultural Sciences, Lanzhou, China. Ethanol absolute (CAS: 64-17-5), sodium hydroxide (CAS: 1310-73-2), and hydrochloric acid (CAS: 7646-01-0) were purchased from Sinopharm Chemical Reagent Co., Ltd. (Shanghai, China).

### 4.2. Flaxseed Gum Extraction

FG extraction was conducted by referring to the method by Sun et al. [47] with appropriate modifications. First, flaxseed was mixed with water in a 1:10 (*w*/*v*) ratio at 50 °C for 2 h and then centrifuged (5000 r/min, 10 min, and 10 °C) to collect the supernatant. This operation was repeated twice. The supernatant was thoroughly mixed with 95% ethanol in a 1:3 (*v*/*v*) ratio, left overnight at 4 °C, and then centrifuged (7500 r/min, 15 min, 10 °C) to collect the precipitate. After the residual ethanol in the precipitate was completely evaporated, it was freeze-dried to obtain flaxseed gum (FG).

### 4.3. Preparation of Gliadin-Flaxseed Gum Composite Particles

Gliadin (50 mg/mL) was fully dissolved in a 70% ethanol solution (*v*/*v*), while FG (5 mg/mL) was completely dissolved by stirring for 2 h (55 °C). A 5 mg/mL GNP reservoir solution was prepared using an anti-solvent method [14] and mixed with 0, 0.125, 0.25, and 0.75 mg/mL of diluted FG solution in equal proportions to prepare the GNP sample solution and composite particles with gliadin-to-FG ratios of 1:0.05, 1:0.1, and 1:0.3 (*w*/*w*), and the pH was adjusted to 4.5 for each group, respectively. The final samples were named as GNP (control), GFG-0.05, GFG-0.1, and GFG-0.3 and the gliadin concentration in the final solution was 2.5 mg/mL.

### 4.4. Turbidity and Viscosity Measurements

Turbidity was determined according to the method by Wang, et al. [48] with appropriate modifications. First, 200 μL of the sample was pipetted onto an enzyme labeling plate, and its absorbance was measured at 600 nm using a multimode microplate reader (VICTOR NivoTM, PerkinElmer^®^, Pontyclun, UK), and the turbidity was expressed in terms of absorbance. The viscosity of the solution was determined using a rotational viscometer (SNB-1, Hifuture, Beijing, China) with the speed set at 60 rpm/s.

### 4.5. Particle Size and Zeta-Potential Measurements

GNP and GFG were diluted to 1 mg/mL using ultrapure water at pH 4.5, and the particle size and ζ-potential were measured using a ZetaSizer Nano analyzer (Zetasizer Pro, Malvern Instruments, Worcestershire, UK).

### 4.6. Microstructure Observations

Freeze-dried GNP and GFG powders were affixed to the conductive adhesive. Subsequently, gold was sputtered onto the sample surface, and the morphology was examined using a scanning electron microscope (ZEISS GeminiSEM 300, Oberkochen, Germany).

### 4.7. Surface Wettability Measurement

The method by Diao et al. [28] was used to measure the surface wettability with minor modifications. The sample powder was pressed into standard flakes measuring approximately 10 mm in diameter and 2 mm in thickness by an automatic tablet press at 10 MPa. Subsequently, 10 μL of water was dropped on the surface, and the droplets were imaged using a CCD camera. A contact angle meter (SDC-200S, SINDIN, Dongguan, Guangdong, China) was then employed to analyze the shape and calculate the contact angle.

### 4.8. Flexibility Measurement

An appropriate amount of freeze-dried sample was dispersed on the surface of the mica plate and the rigidity (Young’s modulus) of the particles was characterized using the PeakForce QNM (Quantitative Nanomechanical Mapping) mode in AFM (Dimension ICON, Bruker, Karlsruhe, Germany).

### 4.9. Visualization of Interfacial Morphology by Cryo-SEM

The interfacial microstructure of foams was visualized using cryo-SEM (SU8010, Hitachi, Ibaraki, Japan). The foam was formed by stirring the samples for 2.5 min via a high-speed electric foamer (MF130, Derlla, Dusseldorf, Germany). After storing for different time, the foams were frozen in liquid nitrogen, followed by sublimating at −70 °C for 12 min and sputter-coating at 5 mA for 30 s. Finally, the morphology was observed by cryo-SEM.

### 4.10. Interfacial Adsorption Behavior Measurement

The surface tension (*γ*) of the samples were measured using a droplet tensiometer (Tracker, Teclis Technologies, Lyon, France) [19,49]. A pear-shaped axisymmetric droplet with a volume of 6 µL was made with a syringe, exposed to a cuvette filled with air, and then the dynamic surface tension was monitored with a video camera for 10,800 s, respectively. The *γ* was obtained according to the Young–Laplace equation. The surface pressure (*π*) was calculated using Equation (1):

(1) $$\pi =\gamma_{0}-\;\gamma$$

where *γ*_0_ indicates the surface tension of solvent, while *γ* is the time-dependent surface tension of the samples.

### 4.11. Interfacial Dilatational Rheology Measurement

The dilatational rheological behavior of the samples at the air/water interface was analyzed using a droplet tensiometer (Tracker, Teclis Technologies, Lyon, France). A sample droplet was formed at the air/water interface and allowed to adsorb for 1000 s, 3000 s, 7200 s, and 10,800 s, respectively. After each adsorption period, amplitude sweeps ranging from 10% to 50% were conducted at a fixed frequency of 0.1 Hz. Additionally, Lissajous plots were fitted with the method reported by Shao et al. [49] and used to analyze the interfacial properties of the samples over time.

### 4.12. Foaming Properties

The foaming capacity (FC) and foam stability (FS) were determined based on the method by Peng et al. [50] and Shao et al. [49]. First, 15 mL of sample solutions (2.5 mg/mL) were placed in a cylindrical flat-bottomed glass container (height 150 mm, inner diameter 30 mm) and homogenized using a shear homogenizer (T25, IKA, Staufen, Germany) at high speed (18,000 r/min) for 2.5 min to generate foam. The foam volume at different time was recorded. *FC* and *FS* were calculated based on Equations (2) and (3):

(2) $$FC(\%)=\frac{V_{2}}{V_{0}}\;\times \;100$$

(3) $$FS\left(\%\right)=\frac{V_{60}}{V_{2}}\;\times \;100$$

where *V*_0_, *V*_2_, and *V*_60_ are expressed as the initial solution volume and the volume of the foam at 2 min and 60 min, respectively.

### 4.13. Statistical Analysis

All experiments were repeated three times and the data were expressed as mean ± standard deviation. ANOVA was performed by SPSS 27.0 (*p* < 0.05) and plots were drawn using Origin 2022.

## Data Availability

The raw data supporting the conclusions of this article will be made available by the authors on request.
